# Slower Elimination of Tofacitinib in Acute Renal Failure Rat Models: Contribution of Hepatic Metabolism and Renal Excretion

**DOI:** 10.3390/pharmaceutics12080714

**Published:** 2020-07-30

**Authors:** Sung Hun Bae, Sun-Young Chang, So Hee Kim

**Affiliations:** Department of Pharmacy, College of Pharmacy and Research Institute of Pharmaceutical Science and Technology, Ajou University, 206 Worldcup-ro, Yeongtong-gu, Suwon 16499, Korea; baezzam@ajou.ac.kr (S.H.B.); sychang@ajou.ac.kr (S.-Y.C.)

**Keywords:** tofacitinib, acute renal failure, gentamicin, cisplatin, pharmacokinetics, hepatic CYP3A1(23), creatinine clearance, renal clearance, nonrenal clearance

## Abstract

Tofacitinib is a Jak inhibitor developed as a treatment for rheumatoid arthritis. Tofacitinib is metabolized mainly through hepatic CYP3A1/2, followed by CYP2C11. Rheumatoid arthritis tends to increase renal toxicity due to drugs used for long-term treatment. In this study, pharmacokinetic changes of tofacitinib were evaluated in rats with gentamicin (G-ARF) and cisplatin-induced acute renal failure (C-ARF). The time-averaged total body clearance (CL) of tofacitinib in G-ARF and C-ARF rats after 1-min intravenous infusion of 10 mg/kg was significantly decreased by 37.7 and 62.3%, respectively, compared to in control rats. This seems to be because the time-averaged renal clearance (CL_R_) was significantly lower by 69.5 and 98.6%, respectively, due to decreased creatinine clearance (CL_CR_). In addition, the time-averaged nonrenal clearance (CL_NR_) was also significantly lower by 33.2 and 57.4%, respectively, due to reduction in the hepatic CYP3A1/2 and CYP2C11 subfamily in G-ARF and C-ARF rats. After oral administration of tofacitinib (20 mg/kg) to G-ARF and C-ARF rats, both CL_R_ and CL_NR_ were also significantly decreased. In conclusion, an increase in area under plasma concentration-time curves from time zero to time infinity (AUC) of tofacitinib in G-ARF and C-ARF rats was due to the significantly slower elimination of tofacitinib contributed by slower hepatic metabolism and urinary excretion of the drug.

## 1. Introduction

**Figure 1:**
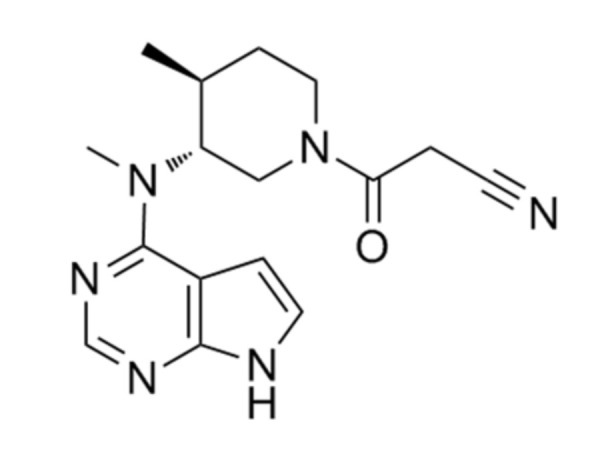
Chemical structure of tofacitinib.

Tofacitinib (Figure 1) was developed as a Jak inhibitor for the treatment of rheumatoid arthritis and is particularly effective when methotrexate is poorly treated [1]. Recently, tofacitinib was approved in 2018 for chronic use to treat moderate to severe ulcerative colitis [2], making it the first Food and Drug Administration (FDA)-approved oral Jak inhibitor [3]. Tofacitinib is currently under clinical trials for various diseases, such as psoriasis [4,5], alopecia [6], atopic dermatitis [7], and ankylosing spondylitis [8].

Pharmacokinetic analysis following oral administration of tofacitinib (10 mg) to healthy volunteers showed a half-life of 3.2 h and a volume of distribution of 87 L [9,10,11]. Approximately 40% of the oral dose bound to plasma protein, 30% of the dose was excreted in the urine as an unmetabolized form, and 70% was metabolized and excreted in the urine as metabolized forms [9,10,11]. Absolute oral bioavailability (*F*) of tofacitinib was found to be approximately 74% [11]. Tofacitinib is primarily metabolized by oxidation and *N*-demethylation in the liver through cytochrome P450 (CYP) 3A4 and CYP2C19 and is further metabolized into glucuronide conjugates [9]. According to a report by Lee and Kim [12], following intravenous, oral, intraportal, intragastric, and intraduodenal administration of 10 mg/kg tofacitinib in male Sprague–Dawley rats, the *F* value was 29.1%, the unabsorbed fraction up to 24 h was 3.16% of the oral dose, the gastric first-pass effect was not significant after intragastric administration of tofacitinib, and 46.1% of the dose administered intraduodenally was metabolized before entering the portal vein. The hepatic first-pass effect was 42% after absorption into the portal vein.

Kidney disease has clinically important significance for patients with rheumatoid arthritis. Kidney disease is correlated with mortality in patients with rheumatoid arthritis [13,14]; therefore, renal impairment can be seen as a predictor of high mortality in patients with rheumatoid arthritis [15]. In addition, rheumatoid arthritis requires long-term treatment, and thus, the possibility of kidney damage is high due to long-term administration of drugs with renal toxicity, such as methotrexate or nonsteroidal anti-inflammatory drugs. Therefore, it is important to accurately measure kidney function in patients with rheumatoid arthritis and necessary to adjust the dose according to the kidney function of the patient. Approximately 36–38% of drugs prescribed in patients with glomerular filtration rate (GFR) < 60 mL/min require dosage adjustment due to pharmacokinetic changes of these drugs [16]. It is known that renal failure occurs in 5–50% of patients with rheumatoid arthritis [17]. Krishnaswami et al. [18] reported that, relative to patients with normal renal function, the mean AUC ratio of tofacitinib for rheumatoid arthritis patients increased progressively with deterioration of renal function. However, no mechanisms for increase of tofacitinib AUC in patients with renal impairment were proposed. There were no reports of tofacitinib showing pharmacokinetic changes associated with hepatic and/or intestinal metabolism, such as CYP protein expression, CYP enzyme activity, or renal function in the acute renal failure model. Since 70% of the dose is metabolized and 30% is excreted in the urine [9,10,11], renal failure seems to significantly impact the metabolism and excretion of tofacitinib as well as its absorption and distribution.

The aim of this study was to evaluate the effects of renal failure on the pharmacokinetics of tofacitinib using gentamicin (G-ARF) and cisplatin-induced acute renal failure (C-ARF) rat models and to report that the increase in AUC of tofacitinib is attributed to the decreases in renal and nonrenal clearances following intravenous and oral administration of tofacitinib to G-ARF and C-ARF rats.

## 2. Materials and Methods

### 2.1. Chemicals

Tofacitinib citrate and hydrocortisone (an internal standard) were obtained from Sigma Aldrich (St. Louis, MO, USA), and ethyl acetate for high-performance liquid chromatography (HPLC) analysis was purchased from J.T. Baker (Phillipsburg, NJ, USA). Gentamicin and cisplatin were obtained from Shin Poong Pharmaceutical (Seoul, Korea) and Tokyo Chemical Industry (Tokyo, Japan), respectively. Heparin and 0.9% NaCl-injectable solution were purchased from JW Pharmaceutical Corporation (Seoul, Korea), and β-cyclodextrin is a product of Wako (Osaka, Japan). Primary antibodies to CYP2B1/2, CYP1A1/2, CYP2D1, CYP2C11, CYP2E1, and CYP3A1/2 were produced by Detroit R&D Inc. (Detroit, MI, USA). β-actin was purchased from Cell Signaling Technology (Beverly, MA, USA). Secondary goat, rabbit, and mouse antibodies were purchased from Bio-Rad (Hercules, CA, USA). All other chemicals and reagents were analysis- or HPLC-grade and used without further purification.

### 2.2. Animals

Sprague–Dawley rats (male, 7 weeks old, weight 200–230 g) were purchased from OrientBio Korea (Seongnam, Korea) and individually managed in a clean room maintained under 12-h light (07:00–19:00)/12-h dark (19:00–07:00) cycles at 22 ± 1 °C with a relative humidity of 50 ± 5% through air purification (Laboratory Animal Research Center of Ajou University Medical Center, Suwon, Korea). All rats were fed with food and water as desired without any restriction. All experimental methods and protocols were carried out according to standard operating procedures with approval by Institutional Animal Care and Use Committee (IACUC No. 2017-0074, 2018) of Laboratory Animal Research Center of Ajou University Medical Center.

### 2.3. Induction of Acute Renal Failure

Rats were randomly divided into three groups: control, G-ARF, and C-ARF rats. Acute renal failure was induced in rats by intraperitoneal injection of gentamicin (100 mg/kg, dissolved in 0.9% NaCl-injectable solution) daily for 8 days [19] or by a single intraperitoneal injection of cisplatin (7.5 mg/kg, dissolved in 0.9% NaCl-injectable solution) [20], while control rats were injected with 0.9% NaCl-injectable solution only. The end times of renal failure induction for pharamcokinetic study of tofacitinib were the next day from the last administration of gentamicin and the sixth day from a single intraperitoneal injection of cisplatin. BUN (Blood urea nitrogen) levels were measured in rats on the last day of induction using a BUN detection kit (Asan Pharmaceutical, Seoul, Korea). Rats with a urea nitrogen level of 36 mg/dL or higher were considered to be acute renal failure-induced [21] and were selected for the study.

### 2.4. Preliminary Study

For preliminary study, plasma samples were collected from control, G-ARF, and C-ARF rats (*n* = 3 per group) to measure total protein, albumin, creatinine, glutamate pyruvate transaminase (GPT), and glutamate oxaloacetate transaminase (GOT) levels (Green Cross Reference Lab, Seoul, Korea). Urine samples were collected for 24 h, and urine volumes and creatinine levels were also measured to estimate the creatinine clearance (CL_CR_). CL_CR_ was calculated by dividing the total amount of creatinine excreted in urine for 24 h by area under the plasma concentration-time curve of creatinine from 0 to 24 h (AUC_0–24 h_), assuming that renal function was stable during the experiment. Whole liver and kidneys were removed from each rat, weighed, partially excised, and soaked in 10% formalin to fix for tissue biopsies.

### 2.5. Intravenous and Oral Administration of Tofacitinib

For oral and intravenous administration, pretreatment and surgical procedures were performed as previously reported [12]. For oral study, the rats were restricted from eating food overnight but water was freely accessible. The next day, after anesthesia with ketamine at a dose of 100 mg/kg, polyethylene 50 tubes (Clay Adams, Parsippany, NJ, USA) was cannulated into the carotid artery for blood collection. For intravenous study, polyethylene 50 tubes were cannulated into the carotid artery and jugular vein for blood collection and drug administration, respectively. After surgery, rats were allowed to rest for 2–3 h to recover from anesthesia and were free in the individual metabolic cage during the experiment.

For intravenous administration, tofacitinib (dissolved in 0.9% NaCl-injectable solution containing 0.5% β-cyclodextrin) was infused for 1 min at a dose of 10 mg/kg via the jugular veins of control (*n* = 6), G-ARF (*n* = 8), and C-ARF (*n* = 7) rats. Blood samples (110 μL) were collected through the carotid artery at 0 (prior to drug infusion), 1 (at the end of drug infusion), 5, 15, 30, 45, 60, 90, 120, 180, 240, 360, 480, 600, and 720 min and were centrifuged at 8000 × *g* for 1 min. Plasma samples were collected and immediately stored in a −80 °C freezer until HPLC analysis of tofacitinib could be performed [22]. After collecting each blood sample, 0.3 mL of heparinized 0.9% NaCl-injectable solution (10 IU/mL) was immediately administered to the carotid artery to prevent blood clotting. At 24 h, the rat’s abdomen was open and the entire gastrointestinal tract was removed, transferred to a beaker with 50 mL of methanol, and cut into small pieces. After mixing the contents in the beaker thoroughly, two 100 μL aliquots of supernatant were taken and stored at −80 °C until HPLC analysis of tofacitinib could be performed [22].

At 24 h after drug administration, urine samples were also collected. The metabolic cage was rinsed with 20 mL of distilled water, which was combined with the 24-h urine sample. The volume of combined urine sample was measured and two 100 μL aliquots of each combined sample were taken and stored in the −80 °C freezer until tofacitinib analysis by HPLC could be performed [22].

For oral administration, tofacitinib at a dose of 20 mg/kg was administered to control (*n* = 8), G-ARF (*n* = 6), and C-ARF (*n* = 8) rats. Blood samples (110 µL) were collected through the carotid artery at 0 (prior to drug administration), 5, 15, 30, 45, 60, 90, 120, 180, 240, 360, 480, 600, and 720 min. At 24 h after drug administration, urine and gastrointestinal tract samples were collected and handled similarly to those in the intravenous study.

### 2.6. Measurement of V_max_, K_m_, and CL_int_

For preparation of hepatic and intestinal microsomes, the experimental processes were similar to a previously reported method [23,24]. Protein concentration in the hepatic and intestinal microsomes was measured using the bicinchoninic acid (BCA) assay. The in vitro metabolic system consisted of microsomes (equivalent to 1 mg protein); 5 µL dimethylsulfoxide containing final tofacitinib concentrations of 1, 2, 5, 10, 20, 50, 100, 200, 300, and 400 µM; and an nicotinamide adenine dinucleotide phosphate hydrogen (NADPH)-generating system (Corning Inc., Corning, NY, USA). The volume of the system was adjusted to 1 mL by adding 0.1 M phosphate buffer (pH 7.4), and the components were incubated in a water-bath shaker at 37 °C with 50 oscillations per min (opm) for 15 min. After this incubation, the reaction was terminated by adding two volumes of acetonitrile. Subsequently, two 50-µL aliquots of each reaction mixture were collected. The kinetic constants, maximum velocity (*V*_max_) and apparent Michaelis–Menten constant (*K*_m_; the concentration at which the rate is one-half of *V*_max_ for the metabolism of tofacitinib) were determined using the Lineweaver–Burk plot [24,25]. The intrinsic clearance (CL_int_) for the metabolism of tofacitinib was determined by dividing *V*_max_ by *K*_m_ [24,25].

### 2.7. Immunoblot Analysis

For immunoblot analysis, microsomal protein samples (20–40 μg protein per lane) were resolved by 10% sodium dodecyl sulfate polyacrylamide gel electrophoresis (SDS-PAGE) gel and the loaded gel was transferred onto a nitrocellulose membrane for 1 h. For immunodetection, blots were incubated overnight with a primary antibody diluted in Tris-buffered saline (TBS) with 0.1% Tween 20 (TBS-T) containing 5% bovine serum albumin (1:2000) at 4 °C with gentle shaking. Subsequently, blots were incubated with secondary antibody conjugated to horseradish peroxide diluted at 1:10,000 with TBS-T containing 5% skim milk for 1 h at room temperature. Protein expression was measured by enhanced chemiluminescence (Bio-Rad) using an Image Quant LAS 4000 Mini (GE Healthcare Life Sciences, Piscataway, NJ, USA). β-actin was used as the internal standard [26]. The density of bands was quantified using ImageJ 1.45s software (NIH, Bethesda, MA, USA).

### 2.8. HPLC Analysis

A 50-μL aliquot of biological sample was mixed with 1 μL hydrocortisone (5 mg/mL); then, 20 μL of 20% ammonia solution was added, mixed with a vortex-mixer (Scientific Industries, Bohemia, NY, USA) for 30 s, and extracted with 750 µL ethyl acetate. The organic layer was collected, evaporated on a thermobath (Eyela, Tokyo, Japan) under a gentle stream of nitrogen gas at 40 °C and redissolved by adding 130 µL 20% acetonitrile, and 50 µL of each reconstituted sample was analyzed by HPLC [12,22].

The concentration of tofacitinib in the biological sample was measured using a Prominence LC-20A HPLC system (Shimadzu, Kyoto, Japan). The reconstituted biological samples were filtered through a 0.45-µm filter (Millipore, Billerica, MA, USA) and analyzed with a reversed-phase column (C_18_; 25 cm × 4.6 mm, 5 µm; Young Jin Biochrom, Seongnam, Korea) using a UV detector at 287 nm. The mobile phase consisted of 10 mM ammonium acetate buffer (pH 5.0) and acetonitrile at a ratio of 69.5:30.5 (*v/v*) with a flow rate of 1.0 mL/min. Tofacitinib and the internal standard were separated at approximately 7.21 and 11.3 min, respectively. The lower limits of quantitation of tofacitinib in rat plasma and urine were 0.01 and 0.1 µg/mL, respectively, and the intraday assay precisions (coefficients of variation) were 3.69–5.88% and 4.21–6.18%, respectively. In addition, interday assay precisions in rat plasma and urine were 5.06% and 5.46%, respectively [22].

### 2.9. Pharmacokinetic Analysis

To estimate pharmacokinetic parameters such as terminal half-life, the apparent volume of distribution at steady state (*V*_ss_), area under plasma concentration-time curves from time zero to time infinity (AUC), mean residence time (MRT), and time-averaged total body (CL), and renal (CL_R_) and nonrenal (CL_NR_) clearances, standard methods [27] were applied using noncompartmental analysis (WinNonlin, Pharsight Corporation, Mountain View, CA, USA). AUCs were calculated using the trapezoidal rule-extrapolation method [28]. The peak plasma concentration (*C*_max_) and time that the plasma concentration was peak (*T*_max_) were directly confirmed from plasma concentration-time curves. To calculate the average values of clearances [29], terminal half-life [30], and *V*_ss_ [31], the harmonic mean method was applied.

### 2.10. Statistical Analysis

The *p* values were estimated using Tukey’s posttest for comparison among three means after analysis of variance (ANOVA) and were considered significant when less than 0.05. All data are expressed as mean ± standard deviation, and a median (ranges) value is used for *T*_max_.

## 3. Results

### 3.1. Induction of Acute Renal Failure

**Figure 2:**
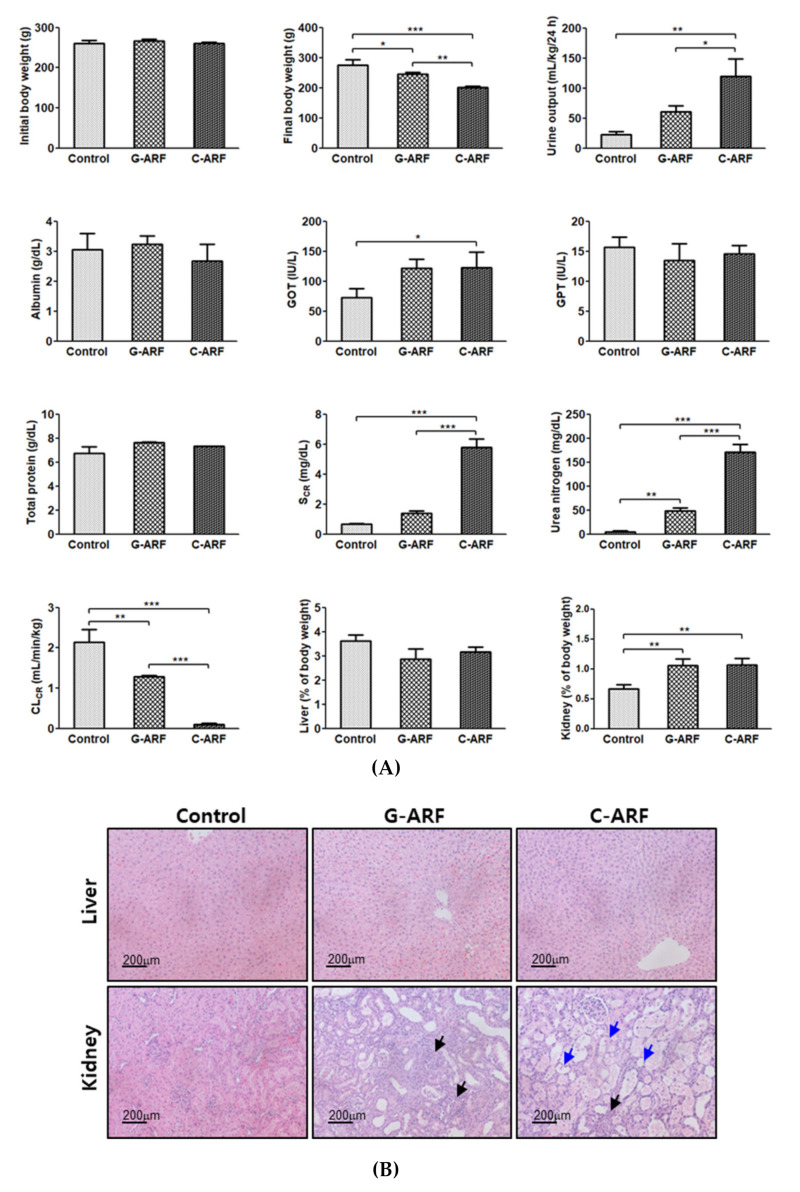
(**A**) The mean values (± standard deviation) of initial and final body weights, 24-h urine output, plasma concentrations of albumin, glutamate oxaloacetate transaminase (GOT), glutamate pyruvate transaminase (GPT), total protein, S_CR_, urea nitrogen, CL_CR_, and relative liver and kidney weights in control, gentamicin (G-ARF) and cisplatin-induced acute renal failure (C-ARF) rats: Bars mean standard deviation. * *p* < 0.05, ** *p* < 0.01, and *** *p* < 0.001; (**B**) Liver and kidney biopsies in control, G-ARF, and C-ARF rats. Black arrows indicate infiltration with immune cells. Blue arrows indicate the tissue damages including tubular necrosis and massive cell death. CL_CR_: creatinine clearance; S_CR_: serum creatinine.

Renal dysfunction was observed in G-ARF and C-ARF rats. Urea nitrogen (898% and 3449% increase, respectively) and creatinine level (111% and 768% increase, respectively) in the blood showed a significant increase and kidney weight (% of body weight) (58.4% and 59.9% increase, respectively) and urine output (172% and 436% increase, respectively) also showed a significant increase, but CL_CR_ was significantly decreased by 39.7% and 95.3% in G-ARF and C-ARF rats, respectively, than in control rats (Figure 2A). A decrease in renal function was also confirmed by kidney microscopy; severe renal damage including tubular necrosis and inflammation was observed in G-ARF and C-ARF rats (Figure 2B). Liver function also appeared to be impaired in G-ARF and C-ARF rats. GOT was significantly increased by 66.2 and 69.0%, respectively; however, no considerable tissue alterations were found in liver microscopy (Figure 2B). In terms of weight gain changes, the body weight gains significantly decreased in G-ARF (8.24% decrease) and C-ARF (23.0% decrease) rats compared to that in control rats (5.75% increase) (Figure 2A). Comparing the two ARF rat models, the C-ARF model showed more severe renal impairment based on urea nitrogen, creatinine, CL_CR_, urine output, kidney weight (% of body weight), and kidney microscopy (Figure 2A,B).

### 3.2. Pharmacokinetics of Tofacitinib After Intravenous Administration

**Figure 3:**
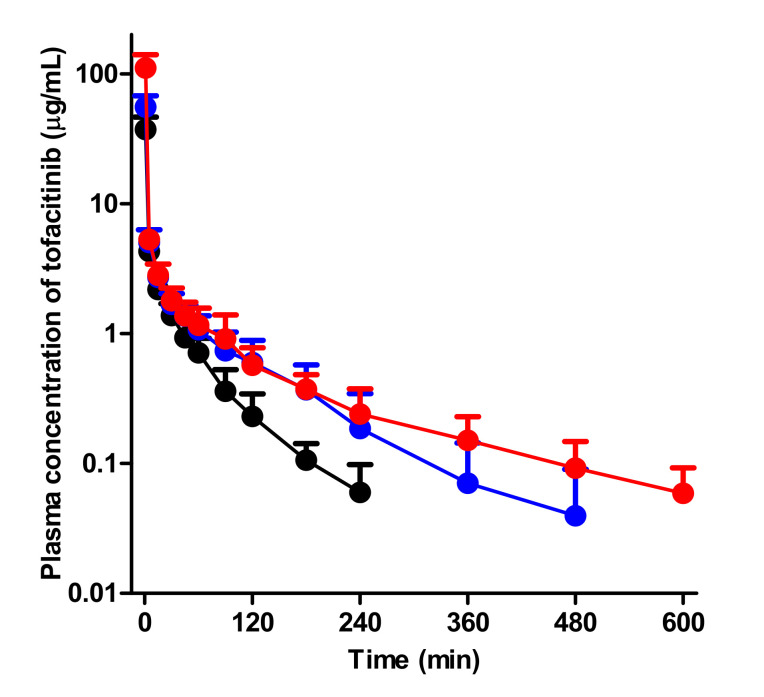
Mean arterial plasma concentration-time curves of tofacitinib after 1-min intravenous infusion at a dose of 10 mg/kg to control (black; *n* = 6), G-ARF (blue; *n* = 8) and C-ARF (red; *n* = 7) rats: Bars represent standard deviation. G-ARF: gentamicin-induced acute renal failure; C-ARF: cisplatin-induced acute renal failure.

After intravenous administration of 10 mg/kg tofacitinib to control, G-ARF, and C-ARF rats, the mean arterial plasma concentration-time curves of tofacitinib declined in a polyexponential fashion for the three groups, with significantly higher plasma levels in rats with G-ARF and C-ARF than in control rats (Figure 3). This resulted in a significantly higher AUC of tofacitinib (64.0 and 163% increase, respectively) than that in control rats (Table 1). The higher AUC of tofacitinib could be due to the significantly lower CL of tofacitinib by 37.7 and 62.3% in rats with G-ARF and C-ARF, respectively (Table 1). A significantly longer terminal half-life (78.7 and 240% increase, respectively) and MRT (96.7 and 154% increase, respectively) of tofacitinib in rats with G-ARF and C-ARF also supports the lower CL of tofacitinib in rats with G-ARF and C-ARF. Lower CL of tofacitinib was due to the slower metabolism of tofacitinib; CL_NR_s of tofacitinib in rats with G-ARF and C-ARF were significantly lower by 33.2 and 57.4%, respectively (Table 1). Tofacitinib excreted in urine as unchanged for 24 h (*Ae*_0–24 h_) was significantly lower by 37.7 and 95.2% in rats with G-ARF and C-ARF, respectively, than that in control rats (Table 1), perhaps due to significantly impaired kidney function in rats with G-ARF and C-ARF. Thus, CL_R_s of tofacitinib were significantly lower by 69.5 and 98.6% in rats with G-ARF and C-ARF, respectively, compared to that in control rats (Table 1). The percentage of dose remaining in the gastrointestinal tract at 24 h (GI_24 h_) was 0.00919–0.195% of the intravenous dose and did not significantly differ among the three groups of rats, suggesting that the contribution of gastrointestinal excretion (including biliary excretion) of tofacitinib to CL_NR_ of tofacitinib was not significant. The *V*_ss_ values were comparable among the three groups (Table 1). Therefore, the significantly lower CL of tofacitinib in rats with G-ARF and C-ARF may be due to slower metabolism and lower renal excretion of tofacitinib than control rats. Plasma concentration and pharmacokinetic parameters of tofacitinib in C-ARF rats were significantly different from those in G-ARF rats (Table 1 and Figure 3) due to more severe renal impairment in C-ARF rats (Figure 2).

### 3.3. Pharmacokinetics of Tofacitinib After Oral Administration

**Figure 4:**
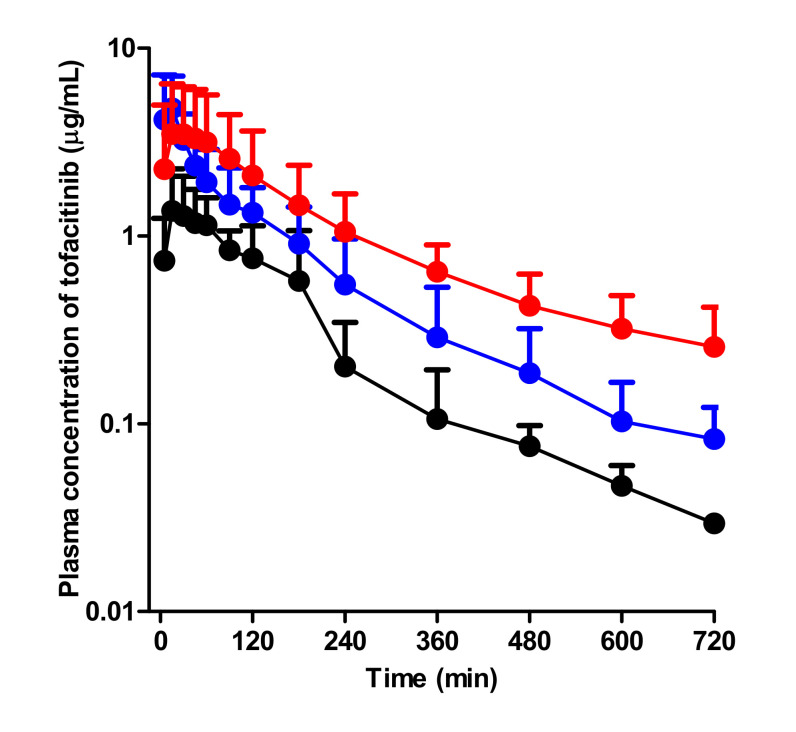
Mean arterial plasma concentration–time curves of tofacitinib after oral administration at a dose of 20 mg/kg to control (black; *n* = 8), G-ARF (blue; *n* = 6), and C-ARF (red; *n* = 8) rats: Bars represent standard deviation. G-ARF: gentamicin-induced acute renal failure; C-ARF: cisplatin-induced acute renal failure.

After oral administration of 20 mg/kg tofacitinib to control, G-ARF, and C-ARF rats, the mean arterial plasma concentration-time profiles of tofacitinib were created and are shown in Figure 4. Relevant pharmacokinetic parameters of tofacitinib were summarized in Table 2.

Absorption of tofacitinib from the gastrointestinal tract occurred rapidly; the plasma concentration of tofacitinib was found at 5 min, the first blood collection time after oral administration in all three groups. Compared to the control rats, G-ARF and C-ARF rats showed higher mean arterial plasma concentration of tofacitinib, resulting in a significant increase in AUC (142 and 247% increase, respectively) and *C*_max_ (198 and 141% increase, respectively) (Table 2). However, the CL_R_ values significantly decreased by 69.8 and 94.0% in G-ARF and C-ARF rats, respectively, due to the significant increase in AUC and a significant decrease in *Ae*_0–24 h_ (22.4 and 81.3% decrease, respectively) in G-ARF and C-ARF rats (Table 2). GI_24 h_ values were 0.231, 1.27, and 0.505% of the oral dose in control, G-ARF, and C-ARF rats, respectively, indicating that absorption of tofacitinib from the gastrointestinal tract was almost complete with no significant difference among the three groups (Table 2). The *T*_max_ values were likewise not significantly different among the three groups. After oral administration, *F* values of tofacitinib were 41.3, 60.7, and 54.3% in control, G-ARF, and C-ARF rats, respectively (Table 2). Plasma concentration and pharmacokinetic parameters of tofacitinib in C-ARF rats, such as AUC, CL_R_, and *Ae*_0–24 h_, were significantly different from those in G-ARF rats due to severe renal impairment in C-ARF rats (Table 2 and Figure 4); this was similar to the results produced by the intravenous study.

### 3.4. Effect of Acute Renal Failure on CYP Enzyme Expression

**Figure 5:**
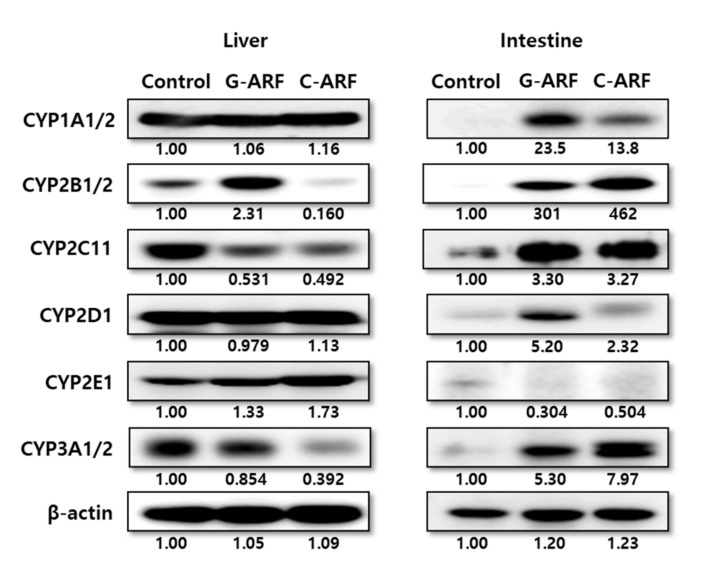
Protein expression of CYP450 isozymes in hepatic and intestinal microsomes in control, G-ARF, and C-ARF rats by immunoblot analyses: ß-actin was used as a loading control. This experiment was performed three times. Band density was measured using ImageJ1.45s software (NIH). G-ARF: gentamicin-induced acute renal failure; C-ARF: cisplatin-induced acute renal failure.

In rats with G-ARF and C-ARF, hepatic and intestinal expression of CYP2B1/2, CYP1A1/2, CYP2D1, CYP2C11, CYP2E1, and CYP3A1/2 were monitored (Figure 5). Immunoblot analysis showed that hepatic expression of CYP2C11 in rats with G-ARF and C-ARF decreased to 53.1 and 49.2% of the level in control rats, respectively, and CYP3A1/2 expression in rats with G-ARF and C-ARF also decreased by 14.6 and 60.8%, respectively, compared to that in control rats. However, CYP2E1 expression in rats with G-ARF and C-ARF increased by 1.33 and 1.73 times, respectively, compared to that in control rats. Expression of CYP1A1/2 and CYP2D1 was comparable among the three groups of rats. Interestingly, protein expression in the intestine showed the opposite trend (Figure 5). The intestinal expression of CYP3A1/2 in rats with G-ARF and C-ARF increased 5.30 and 7.97 times, respectively, and CYP2C11 expression also increased by 3.30 and 3.27 times, respectively, compared to those in control rats. Other CYP protein expressions except CYP2E1 also increased in the intestine of G-ARF and C-ARF rats.

### 3.5. Measurement of V_max_, K_m_, and CL_int_ of Tofacitinib in Hepatic Microsomes

**Figure 6:**
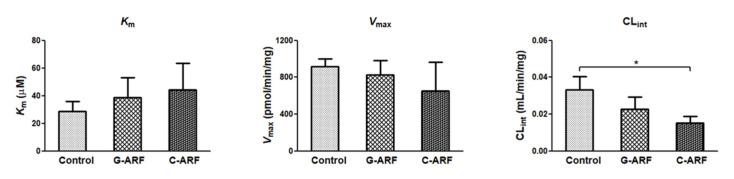
Measurement of *V*_max_*, K_m_,* and CL_int_ for the disappearance of tofacitinib in hepatic microsomes of control, G-ARF, and C-ARF rats (*n* = 3 per group): This experiment was performed three times, and data are expressed as mean ± standard deviation. Bars represent standard deviation. * *p* < 0.05. *V*_max_: maximum velocity; *K*_m_: apparent Michaelis–Menten constant, the concentration at which the rate is one-half of *V*_max_ for the metabolism of tofacitinib; CL_int_: intrinsic clearance; G-ARF: gentamicin-induced acute renal failure; C-ARF: cisplatin-induced acute renal failure.

In rats with G-ARF and C-ARF, the *V*_max_ values for the disappearance of tofacitinib in the hepatic microsomal protein decreased by 9.52 and 28.7%, respectively, but were not significantly different compared to that in control rats (Figure 6). *K*_m_ values were comparable among the three groups (Figure 6). However, CL_int_ for the disappearance of tofacitinib in the hepatic microsomal protein was significantly lower (54.4% decrease) in rats with C-ARF compared to that in control rats (Figure 6), suggesting that disappearance of tofacitinib could be slower in C-ARF rats. CL_int_ was also lower (31.1% decrease) but not significantly different in G-ARF rats compared to that in control rats. Taken together, our data indicate that G-ARF or C-ARF affect hepatic function to inhibit the expression of CYP3A1/2 and CYP2C11, resulting in slower metabolism of tofacitinib.

## 4. Discussion

To establish acute renal failure, gentamicin and cisplatin were chosen. Gentamicin, a representative aminoglycoside antibiotic, induces moderate and reversible acute renal failure [32], while cisplatin, a chemotherapeutic drug, causes more severe and irreversible acute renal failure [33]. Both drugs accumulate in the renal tubule; produce reactive oxygen species (ROS) such as superoxide anion, hydrogen peroxide, and hydroxyl radical; and result in the induction of tubular necrosis and/or apoptosis [34,35,36]. The impaired renal function caused by these drugs was fully demonstrated, but liver damage did not seem to be serious in our preliminary study. Induction of acute renal failure by gentamicin and cisplatin was confirmed not only by a significant decrease in weight gain and CL_CR_ but also by significant increases in 24-h urine output and plasma levels of urea nitrogen and creatinine than those in control rats. Renal biopsy also demonstrated the induction of acute renal failure.

The contribution of gastrointestinal excretion (including biliary excretion) as unchanged tofacitinib to CL_NR_ of the drug seems to be nearly negligible. The GI_24 h_ values were negligible in control, G-ARF, and C-ARF rats, i.e., less than 0.195% of the intravenous dose in the three groups (Table 1). This lower GI_24 h_ did not appear to be caused by chemical or enzymatic degradation of tofacitinib in the rat’s gastrointestinal tract; tofacitinib was stable when incubated for 24 h in various buffers of pH 2–10 [22] and in the gastric juice of rats (pH 3.5) (data not shown). Furthermore, according to a report by Lee and Kim [12], when 10 mg/kg tofacitinib was intravenously administered to rats (*n* = 3) after bile duct cannulation, biliary excretion of unchanged tofacitinib for 24 h was 0.703% of the intravenous dose, a nearly negligible contribution to CL_NR_ of tofacitinib. Therefore, the CL_NR_ value shown in Table 1 may represent the metabolic clearance of tofacitinib.

The AUCs of tofacitinib were not dose proportional after intravenous doses over 20 mg/kg and oral doses over 50 mg/kg were administrated [12]. A dose of 10 mg/kg of tofacitinib was chosen for the intravenous study, and 20 mg/kg of tofacitinib was selected for the oral study. After intravenous administration of tofacitinib to G-ARF and C-ARF rats, its AUCs were significantly higher, possibly as a result of significantly slower CL than in control rats. The lower CL of tofacitinib was attributable to significantly decreased CL_R_ and CL_NR_ of the drug in rats with G-ARF and C-ARF than in controls. The lower CL_R_ may have been due to both significantly lower *Ae*_0–24 h_ and higher AUCs in G-ARF and C-ARF rats. The higher AUC in rats with G-ARF and C-ARF was due to lower *Ae*_0–24 h_ and lower CL_NR_ than those in control rats. The lower *Ae*_0–24 h_ in G-ARF and C-ARF rats could have been due to impaired kidney function. Tofacitinib did not show a urine flow rate-dependent timed-interval CL_R_ in rats; a straight line was not found between 1/timed-interval CL_R_ of tofacitinib and 1/urine flow rate among the three groups [37]. Greater urine output did not result in higher *Ae*_0–24 h_ of tofacitinib, indicating that tofacitinib was not predominantly reabsorbed in the renal tubule. The significantly greater urine output in rats with G-ARF and C-ARF was because reabsorption of water was decreased in the renal tubule due to decrease in protein expression of aquaporins caused by gentamicin and cisplatin [38]. However, *Ae*_0–24 h_ of some drugs showed a urine flow rate-dependent timed-interval CL_R_ in rats; the lower the urine output, the lower the *Ae*_0–24 h_ [37], which resulted in a straight line between 1/timed-interval CL_R_ and 1/urine flow rate in both control and uranyl nitrate-induced acute renal failure (U-ARF) rats [23,39].

The CL_R_ values of tofacitinib were estimated from free (unbound to plasma proteins) fractions in plasma; the values thus estimated were 5.99, 1.83, and 0.0856 mL/min/kg for control, G-ARF, and C-ARF rats, respectively, based on 20.7% plasma protein binding of tofacitinib measured by equilibrium dialysis [22]. The CL_R_ values of tofacitinib were faster than their respective CL_CR_s in control and G-ARF rats, but CL_R_s of tofacitinib and CL_CR_ in C-ARF rats were comparable each other, suggesting that tofacitinib is mainly excreted in urine via active secretion for control and G-ARF-rats [12,18,24] and in glomerular filtration for C-ARF rats. This was also supported by control and U-ARF rats; CL_R_ of metformin and chlorzoxazone were faster than CL_CR_ in control rats, but CL_R_ of both drugs and CL_CR_ were comparable in U-ARF rats, and thus both drugs were mainly excreted in urine via active secretion for control rats and glomerular filtration for U-ARF rat [23,39]. As shown in the results, both gentamicin and cisplatin induced acute renal failure by tubular necrosis through ROS production, but cisplatin induced more severe renal failure and seemed to completely inhibit the function of active secretion [40].

Based on the AUC difference between intravenous and intraportal administration of tofacitinib to rats, the first-pass metabolism of tofacitinib by the liver after reaching the portal vein was approximately 42.0% [12]. Therefore, tofacitinib has a characteristic with an intermediate hepatic extraction ratio and its hepatic clearance could be changed by both the hepatic CL_int_ and the hepatic blood flow rates [41]. Thus, a significantly lower CL_NR_ of tofacitinib when administered intravenously to rats with G-ARF and C-ARF could have been due to a significantly lower CL_int_ for the elimination of tofacitinib in the liver. The reduced protein expressions and activities of the hepatic CYP3A1/2 and CYP2C11 subfamily could have been responsible for the lower hepatic CL_int_ in G-ARF and C-ARF rats. Similar results with regard to changes in hepatic CYP3A1/2 and/or CYP2C11 isozymes have been reported in acute renal failure rats induced by glycerol, bilateral ureter ligation, and nephrectomy [42]. Surgically induced chronic kidney disease rat models also showed lower levels of hepatic CYP3A and CYP2C subfamilies compared to sham-operated control rats [43]. Consistent with our result in renal failure rat models, the CYP3A subfamily also decreased in patients with end-stage renal failure [44].

After intravenous administration of tofacitinib to control, G-ARF, and C-ARF rats, its *V*_ss_ values were not significantly different among the three groups of rats, since the free fractions of tofacitinib in the plasma from control, G-ARF and C-ARF rats were comparable (data not shown). Similar results were also reported for cyclosporin [45] in G-ARF rats and tacrolimus [46] in C-ARF rats, whereas the *V*_ss_ of metformin [23]; omeprazole [26]; and DA-1131, a new carbapenem [47] in U-ARF rats, significantly increased compared to that in control rats, which was due to the increase in free fraction of the drugs.

After oral administration of tofacitinib to rats with G-ARF and C-ARF, the AUC values were also significantly higher than in control rats. The absorption of tofacitinib from the gastrointestinal tract was almost complete among all three groups; GI_24 h_ values were less than 1.27% of oral dose for control, G-ARF, and C-ARF rats. Therefore, absorption is not a factor for the higher AUCs in rats with G-ARF and C-ARF. However, decreased absorption after oral administration of azosemide [48]; oltipraz [49]; and YJA-20379-8, a new proton pump inhibitor [50], to rats with U-ARF has been reported. Although the expression of CYP3A1/2 and CYP2C11 in the intestine markedly increased in rats with G-ARF and C-ARF compared to those in control rats, AUC of tofacitinib increased in rats with G-ARF and C-ARF. The CL_int_ for the disappearance of tofacitinib in the intestinal microsome was not measured in this study because the active site of CYP3A1/2 and CYP2C11 in the intestine was sensitive and very unstable [51]. It has been reported that CYP3A activity in the intestine was increased in renal failure models induced by cisplatin, glycerol, bilateral ligation, or nephrectomy [42]. The AUC increase in rats with G-ARF and C-ARF might be because active secretion of tofacitinib was reduced by tubular necrosis caused by gentamicin and cisplatin. Thus, tofacitinib accumulated in the body, resulting in significantly lower *Ae*_0–24 h_ of oral dose and significantly lower CL_R_ along with increased AUCs in rats with G-ARF and C-ARF than in control rats (Table 2). In addition, considering that approximately 21.3% of the oral dose was metabolized in the liver of control rats after oral administration of tofacitinib [12], the hepatic first-pass effect seemed to decrease in rats with G-ARF and C-ARF after absorption of tofacitinib into the portal vein. This was likely due to decreased hepatic enzyme activity, and protein expression of CYP3A1/2 and CYP2C11 in rats with G-ARF and C-ARF, which also contributed to the increase in AUC of tofacitinib after oral administration of the drug.

Consistent with our data in renal-failure rat models, results of a previous study showed that CYP3A subfamily decreased in patients with end-stage renal failure [44]. In patients with severe renal impairment, the plasma concentration of tofacitinib was significantly increased and, thus, the AUC of tofacitinib in these patients was higher than twice that in normal healthy subjects, suggesting that the reduction of tofacitinib dosage is recommended in patients with severe renal failure [18]. Because the renal excretion of tofacitinib was different between human and rats (approximately 30% of oral tofacitinib in human [9] and 6.21% of oral dose in rats), it is difficult to clearly conclude the clinical significance of the rat’s results, but it seems clear that the increase of AUC in the renal failure state was due to slower hepatic metabolism and smaller urinary excretion of the drug. Our study could be applied to drug–drug interactions in clinical practice when administered in combination with CYP3A4 and CYP2C19 inhibitors, such as itraconazole and erythromycin, which may result in an increase in plasma concentration of tofacitinib by reduced nonrenal elimination of tofacitinib. Therefore, it is necessary to consider the dose reduction of tofacitinib when AUC increases twice or more.

## 5. Conclusions

After intravenous administration of tofacitinib to G-ARF and C-ARF rats, its AUC was significantly higher than that in control rats due to a significantly lower CL_NR_ (due to a decrease in the protein expressions of the hepatic CYP3A1/2 and CYP2C11 subfamily) and CL_R_ (due to a significantly lower CL_CR_ by an impaired kidney function) than in control rats. A reduced dosage of tofacitinib could be considered in patients with renal impairment based on their level of renal dysfunction.

## Figures and Tables

**Table 1:** Mean (±standard deviation) pharmacokinetic parameters of tofacitinib after 1-min intravenous infusion at a dose of 10 mg/kg to control, G-ARF, and C-ARF rats.

Parameters	Control (*n* = 6)	G-ARF (*n* = 8)	C-ARF (*n* = 7)
Body weight (g) ^a^	280 ± 19.0	251 ± 21.3	188 ± 10.2
Terminal half-life (min) ^b^	39.4 ± 11.3	70.4 ± 29.6	134 ± 40.9
AUC (μg∙min/mL) ^a^	264 ± 45.4	433 ± 90.0	693 ± 105
MRT (min) ^c^	27.2 ± 10.4	53.5 ± 30.2	69.1 ± 39.6
CL (mL/min/kg) ^d^	39.0 ± 7.97	24.3 ± 6.95	14.7 ± 2.29
CL_R_ (mL/min/kg) ^e^	4.75 ± 1.28	1.45 ± 1.54	0.0679 ± 0.0917
CL_NR_ (mL/min/kg) ^f^	34.3 ± 6.77	22.9 ± 5.54	14.6 ± 2.26
*V*_ss_ (mL/kg)	1042 ± 402	1174 ± 519	1002 ± 558
*Ae*_0–24 h_ (% of dose) ^a^	9.51 ± 0.879	5.92 ± 2.76	0.458 ± 0.626
GI_24 h_ (% of dose)	0.153 ± 0.306	0.00919 ± 0.0225	0.195 ± 0.157

*Ae*_0–24 h_: percentage of the dose excreted in the 24-h urine; AUC: total area under the plasma concentration–time curve from time zero to time infinity; C-ARF: cisplatin-induced acute renal failure; CL: time-averaged total body clearance; CL_NR_: time-averaged nonrenal clearance; CL_R_: time-averaged renal clearance; G-ARF: gentamicin-induced acute renal failure; GI_24 h_: percentage of the dose remaining in the gastrointestinal tract (including its contents and feces) at 24 h; MRT: mean residence time; *V*_ss_: apparent volume of distribution at steady state. ^a^ Control is significantly different (*p* < 0.01) from C-ARF and G-ARF. ^b^ Control is significantly different from C-ARF (*p* < 0.001) and G-ARF (*p* < 0.01). ^c^ Control is significantly different from G-ARF (*p* < 0.05). ^d^ Control is significantly different from G-ARF and C-ARF (*p* < 0.001). G-ARF and C-ARF were significantly different (*p* < 0.05). ^e^ Control is significantly different from G-ARF and C-ARF (*p* < 0.001). ^f^ Control is significantly different from G-ARF (*p* < 0.01) and C-ARF (*p* < 0.001). G-ARF and C-ARF were significantly different (*p* < 0.01).

**Table 2:** Mean (±standard deviation) pharmacokinetic parameters of tofacitinib after oral administration at a dose of 20 mg/kg to control, G-ARF, and C-ARF rats.

Parameters	Control (*n* = 8)	G-ARF (*n* = 6)	C-ARF (*n* = 8)
Body weight (g) ^a^	264 ± 26.3	206 ± 14.9	174 ± 11.7
AUC (μg∙min/mL) ^b^	217 ± 22.3	525 ± 178	752 ± 420
*C*_max_ (μg/mL) ^c^	1.74 ± 0.606	5.18 ± 2.59	4.20 ± 3.03
*T*_max_ (min)	71.9 ± 64.7	30.8 ± 43.9	41.3 ± 36.5
CL_R_ (mL/min/kg) ^a^	5.66 ± 1.03	1.71 ± 0.871	0.300 ± 0.495
*Ae*_0–24 h_ (% of dose) ^d^	6.21 ± 1.12	4.82 ± 3.06	1.16 ± 1.37
GI_24 h_ (% of dose)	0.231 ± 0.235	1.27 ± 1.72	0.505 ± 0.535

*Ae*_0–24 h_: percentage of the dose excreted in the 24-h urine; AUC: total area under the plasma concentration–time curve from time zero to last time; C-ARF: cisplatin-induced acute renal failure; CL_R_: time-averaged renal clearance; *C*_max_: peak plasma concentration; G-ARF: gentamicin-induced acute renal failure; GI_24 h_: percentage of the dose remaining in the gastrointestinal tract (including its contents and feces) at 24 h; *T*_max_: time that the plasma concentration was peak. ^a^ Control is significantly different from G-ARF and C-ARF (*p* < 0.001). G-ARF and C-ARF were significantly different (*p* < 0.05). ^b^ Control is significantly different from C-ARF (*p* < 0.01). ^c^ Control is significantly different from G-ARF (*p* < 0.05). ^d^ C-ARF is significantly different from control (*p* < 0.001) and G-ARF (*p* < 0.05).
